# Health Technology Assessment for Vaccines Against Rare, Severe Infections: Properly Accounting for Serogroup B Meningococcal Vaccination's Full Social and Economic Benefits

**DOI:** 10.3389/fpubh.2020.00261

**Published:** 2020-07-10

**Authors:** Andrew Stawasz, Liping Huang, Paige Kirby, David Bloom

**Affiliations:** ^1^Data for Decisions, LLC, Waltham, MA, United States; ^2^Harvard Law School, Cambridge, MA, United States; ^3^Pfizer Inc., Collegeville, PA, United States; ^4^Harvard T.H. Chan School of Public Health, Boston, MA, United States

**Keywords:** vaccination, immunization, meningococcal, meningitis B, health technology assessments, cost effectiveness

## Abstract

The high price of new generations of vaccines relative to their predecessors has become an important consideration in debates over whether the benefits of the new vaccines justify their costs. An increasingly central line of inquiry in the literature on valuing vaccination surrounds accounting for the full social and economic benefits of vaccination. This paper applies this emerging perspective to the particular case of vaccination against serogroup B meningococcal disease (MenB). We explore key issues involved in health technology assessments of MenB vaccination, which have led to pronounced heterogeneity in evaluation methods and recommendation outcomes across countries such as France, Germany, the US, and the UK. Accounting for typically neglected sources of socioeconomic benefit could potentially impact recommendation and reimbursement decisions. We propose a taxonomy of such benefits built around four dimensions: (i) internalized health benefits, (ii) internalized non-health benefits, (iii) externalized health benefits, and (iv) externalized non-health benefits. This approach offers a systematic, comprehensive evaluation framework that can be used in future assessment of MenB vaccines as well as other health technologies.

## Introduction

Considerable discussion has surrounded the rising costs of vaccines in recent years (1). Earlier generations of vaccines were affordable and highly cost-effective, which made including them in publicly financed immunization programs an easy decision for governments. But newer vaccines—including those against serogroup B meningococcal disease (MenB)—are more expensive, and those for rare diseases like MenB can have less certain benefits due to data scarcity. Vaccine policymakers therefore must and do scrutinize them more closely.

Recent literature, however, highlights wide gaps between what policymakers typically count as vaccine benefits and the full benefits that vaccines actually confer (2–20). Failure to consider substantial portions of vaccines' full benefits can lead to undervaluing vaccines, which can in turn lead to ill-informed vaccine development, recommendation, and reimbursement decisions. Unnecessary preventable health burdens and their downstream social and economic consequences are an unfortunate possibility under circumstances of such undervaluation.

Along these lines, traditional economic evaluations of MenB and other meningococcal vaccines typically ignore or undercount several plausibly substantial benefits. Such traditional evaluations can therefore distort vaccine policymaking decisions.

As this paper explains in greater detail, the broader benefits that the literature fails to capture include, but are not limited to, productivity gains (both paid and unpaid work) and leisure gains for would-be patients and their caregivers, the insurance value from protecting risk-averse individuals against mortality and morbidity risks, societal preferences for preventing especially severe illnesses like MenB, reductions in socioeconomic inequality, and mitigating contributions to macrosocial public health challenges such as nosocomial infections.

### Goals and Organization of This Paper

This paper expands upon the literature's taxonomy of vaccination benefits to include socioeconomic aspects often unaccounted for by conventional, health-centric economic evaluation methods. Our exploration of this concept aims to broaden analysts' and policymakers' understanding of the scope and probable magnitude of vaccination benefits in general. This paper focuses on valuation in the unique context of MenB, though many of the concepts and issues discussed are broadly applicable to meningococcal disease in general and other infectious diseases as well.

We first provide a brief epidemiological background on MenB and outline the key technical issues involved in health technology assessments (HTAs) of this disease. We then discuss how such unresolved issues have resulted in pronounced heterogeneity in evaluation methods and outcomes across countries. The UK's experience with economic evaluations of MenB illustrates how adopting a broad benefits perspective can help resolve some of these issues and influence recommendation decisions. Supplementing those benefits considered by the UK, we present a comprehensive framework for conceptualizing the full value of vaccination from a societal perspective. We conclude by exploring the implications of this new framework for MenB-related policy discussions and future research.

### Background on Meningococcal Disease and MenB

Meningococcal disease (MD) comprises infections caused by the bacterium *Neisseria meningitidis*. This pathogen can be further divided into distinct serogroups, among which A, C, X, Y, W, and B are the most common (21–23). All serogroups can harmlessly colonize the nasopharynx; in some cases, it can attach to the surface of mucosal cells in the nasopharynx and cause pneumonia and other usually mild, localized infections. However, if the bacterium moves to infect a normally sterile part of the body, such as the bloodstream (sepsis) or the brain, spinal cord, or surrounding fluid (meningitis), much more dangerous infections can occur (23, 24). These kinds of infections are collectively known as invasive meningococcal disease (IMD) (25).

IMD is primarily concentrated in two age groups: those under 1 year old and adolescents or young adults (26, 27). The pathogen is transmitted from person-to-person through respiratory or throat secretions (saliva) (28), increasing the risk of IMD for groups that live in close quarters and have prolonged contact. Meningococcal carriage occurs predominantly in adolescents (29–31), and in many countries including the United States, IMD is a particular threat to young adults given the frequent, close contact among students on college campuses (31–33). Other close-contact groups at disproportionate risk of IMD outbreaks include those living in military barracks (31, 34) and Hajj pilgrims (31, 35, 36).

The global distribution and incidence of IMD varies over time and by region and serogroup. For instance, the meningitis belt of sub-Saharan Africa is a region characterized by relatively high IMD incidence rates (10–1,000 cases per 100,000 population) (23) and frequent outbreaks. In this region, serogroup A was responsible for the majority of cases between 2007 and 2009 while serogroup W has been dominant since approximately 2010 (followed by serogroups A, X and, to a lesser extent, C) (37, 38). IMD incidence is comparatively lower in North America and Europe (0.5–0.9 cases per 100,000 population) (23), where serogroup B is one of the predominant serogroups (along with serogroup C) (26, 39). Meningococcal serogroup B is the focus of this paper.

Similar to other serogroups, perhaps the two most defining features of serogroup B (MenB) are its rarity and severity. MenB is a notably uncommon disease. Even in countries where B is the dominant meningococcal serogroup, MenB incidence in recent years is low compared with that of other major diseases. For example, over the period 1991–2011, MenB disease incidence in Canada ranged from 0.1–0.9 per 100,000 population, and in 2016 the incidence of MenB in the US was only 0.05 per 100,000 (40, 41). For reference, the HIV incidence in Canada and the US in 2016 was 6.4 and 12.3 per 100,000, respectively (42, 43).

If MenB's rarity tends to decrease the disease's burden, its severity and unpredictability tend to have the opposite effect. MenB is a severe disease by almost any measure. During the acute phase of MenB, the median health utility index on the worst day of disease is −0.056, implying a health state worse than death (44). Moreover, 10–20% of MD infections in general end in death, a figure that climbs to 40% when the infection results in sepsis (45). This case fatality rate is many times greater than those of other burdensome infections like severe dengue (2.5%), childhood pneumonia (1%), H1N1 pandemic influenza (<1%), and malaria (<1%) (46–49).

Furthermore, the consequences of the disease can be devastating for MenB survivors as its associated burdens continue long after the acute phase; in fact, many IMD survivors experience permanent disability, which can include but is not limited to:

- Severe neurologic, cognitive, or behavioral disability;
- Visual impairment;
- Amputation;
- Hearing loss;
- Seizures;
- Skin necrosis and scarring;
- Chronic pain;
- Renal dysfunction or failure; and
- Anxiety and depression (50).

A major case-control study by Viner et al. examining the UK's National Meningococcal Registry finds that about one tenth of children who survive MenB develop a highly debilitating disability, while more than a third have at least one deficit in physical, cognitive, or psychological functioning. The same study finds an association between surviving MenB and worse outcomes related to IQ, executive function, psychological health, and memory (51).

Exacerbating MenB's severity are the difficulties surrounding diagnosis in its early stages. Initial symptoms of MenB include fever, irritability, nausea or vomiting, decreased appetite, headache, and abnormal skin color. But within hours, these may rapidly give way to more alarming symptoms such as photophobia, confusion or delirium, bulging fontanelle (among young infants), seizures, or unconsciousness (52). Because of MenB's severity, all suspected cases should result in hospital admission. But MenB's early, non-specific symptoms create uncertainty, and as a result physicians do not always accurately diagnose MenB and begin antibiotic treatment before it progresses to more serious stages. One study in the UK finds that only 51% of all MD patients are actually admitted as inpatients after the first consultation, implying that physicians in the UK fail to diagnose MD about half the time (52). The same UK study shows that children reach advanced disease stages or death in as little as 24 h after disease onset (52). Thus, the difficulty in diagnosing MD early plays a large role in its severe outcomes.

Finally, the incidence of MenB within a population is highly unpredictable (53). The majority of MenB cases (98%) are sporadic (that is, not related to an ongoing outbreak), occur worldwide, and can strike unexpectedly in otherwise healthy individuals (54–56). Further, while MD incidence is generally concentrated in infants and adolescents (26, 27), individuals of any age can contract the disease, including the elderly (57). A surveillance analysis of MenB in Australia between 2006 and 2015, for instance, found the share of confirmed cases to be distributed across age buckets of <12 months (19%), 1–4 years (19%), 5–19 years (29%), 20–24 years (10%), and over 25 years of age (23%) (58). From an individual perspective, it is therefore extremely difficult to predict vulnerability to the disease, which can strike even young adults in their prime with potentially devastating consequences, including death.

## Key Challenges in Health Technology Assessments of MenB

MenB's rarity and severity, and the underlying tension between them, present unique challenges for health technology assessors scrutinizing the investment value of MenB vaccines. On the one hand, the severity of MenB tends to raise the value of MenB vaccination. The associated health burdens lead to substantial economic costs: mean healthcare costs in the US in the first year after disease exceed $30,000 without long-term complications and approach $100,000 with such complications (59). Martinón-Torres reports that lifelong rehabilitation and treatment costs for long-term IMD sequelae can be enormous—amounting to millions of US dollars. The same review observes that many of IMD's cognitive, psychosocial, and psychiatric burdens are systematically overlooked, implying that the true burden is likely much higher (45).

IMD's severity costs also relate to the harms stakeholders other than the disease victim experience. Al-Janabi et al. find that the quality-adjusted life year (QALY) loss among families and caregivers of those with meningitis sequelae is 16% of the loss experienced by the disabled person, largely owing to increased rates of anxiety and depression (60). Conservatively assuming that each IMD patient has only one caregiver, these findings imply that IMD's total health burden is about 1.16 times its already extreme burden to the patient. Of course, this burden increases further in the case of more household contacts and caregivers.

Moreover, the substantial costs of IMD outbreaks can burden entire economies. These costs relate not only to the greater number of costly IMD treatments, but also to the costs of managing and containing the outbreaks. Outbreak control costs include costs related to procuring and delivering vaccines or chemoprophylaxis therapy rapidly and to utilizing more medical staff, public health workers, and volunteers (61).

Despite IMD's high severity costs, however, its rarity is so pronounced that cost-effectiveness analyses (CEAs) of MenB vaccines often disfavor the vaccines unless vaccine costs are low (62–65). This is in large part because few infections are present for the vaccine to prevent, either directly or indirectly through herd effects. This rarity also means that collecting reliable data on MenB or MenB vaccination's impact is difficult. Only small sample sizes are available for study (66), making fundamental vaccine-related parameters—such as vaccine efficacy, the rate of vaccine efficacy waning, and the magnitude of herd effects (67)—highly uncertain (68). The latter parameter is especially critical as it has been shown to influence the cost-effectiveness of adolescent MenB vaccination (69).

These factors—MenB vaccination's potential cost-ineffectiveness and many highly uncertain parameters—can chill decisions to recommend, reimburse, or invest in research and development for MenB vaccines despite the disease's severity. This same tension between rarity and severity exists for other infectious diseases as well. To the extent that MenB represents other rare, severe diseases, this situation could also create the impression that vaccines against other such diseases are also unworthy of recommendation, reimbursement, or research and development (70).

### Heterogeneity in HTAs and Recommendation Policies

There is no clear consensus in the literature on how properly to resolve the assessment issues surrounding MenB's rarity and severity. As a result, there exists pronounced heterogeneity in MenB vaccination evaluations and recommendations across countries and risk groups. Examining the differences in HTAs between France, Germany, the US, and the UK, for example, well illustrates the absence of a systematic, encompassing approach to measuring the benefits of MenB vaccination.

In France, policymakers recommend MenB vaccination only for those in certain endemic or hyperendemic situations. It is not recommended routinely for any nationwide population, nor is it recommended prophylactically in response to a MenB outbreak (71). Many health technology decisions in France are made without an accompanying economic analysis. The Haute Autorité de Santé (HAS)—or National Authority for Health—is assigned to produce “medico-economic opinions,” but conducting pharmacoeconomic studies or abiding by their conclusions for individual vaccines is not mandatory (72). While the HAS in France did consider an economic analysis of routine MenB vaccination in making its decision not to recommend the vaccine, the analysis considered only direct health costs (73). That French authorities performed such a health economic analysis is laudable; however, this analysis is non-mandatory, its outcomes are non-binding, and its considered list of benefits is far from comprehensive, rendering its conclusions therefore at least suspect.

Germany's situation resembles that of France in that routine MenB vaccination is not clinically recommended as part of the national immunization program (74), and the results of pharmacoeconomic assessments are non-binding on decisions regarding clinical vaccine recommendations (75). These analyses' main role surrounds setting maximum reimbursement prices for approved vaccines (76). In Germany, however, performing and reviewing these assessments is mandatory as opposed to merely encouraged (75, 76). Further, one prominent CEA of routine MenB vaccination in Germany did go beyond narrow benefits, including broad benefits such as caregiver productivity gains during the acute disease phase. Despite this, the analysis found that the vaccine is likely to be cost-ineffective (62). Including productivity gains is a step toward a broader benefits framework; but, as with the French analysis, this analysis falls well-short of comprehensively counting all important benefit sources. This non-comprehensive approach again calls into question the conclusiveness of such results.

In the US, the Advisory Committee on Immunization Practices (ACIP) has two categories of recommendation for MenB vaccination. A strong “Category A” recommendation is only for those at increased risk of disease—for example, patients with anatomic or functional asplenia or researchers who work with meningococcal bacteria directly—and those in institutions or areas experiencing an active outbreak (77). A weaker “Category B” recommendation, which merely indicates that target populations *may* receive MenB vaccination, applies to all other individuals aged 16–23, including college students and other at-risk populations (77). No recommendation exists for young children. HTAs in the US are similar to those in France in that they have focused primarily on short- and long-term health costs. Assessors in the US have acknowledged other considerations such as herd effects, outbreak-related costs, and societal preferences for preventing severe diseases, but have found data limitations an obstacle to including these in CEAs of MenB (78). Ultimately, US policymakers are sensitive to MenB's rarity, but not to capturing the full burden of its severity, which has resulted in only a weak recommendation for routine vaccination. Failure to consider the vaccine's full benefits again renders this non-recommendation suspect.

A common theme therefore underlies the recommendation policy decisions of each of these countries: HTAs routinely fail to capture a comprehensive spectrum of socioeconomic benefits that could reveal important additional sources of value for MenB vaccination programs. A full benefits approach that adequately considers the extreme severity of MenB—such as that embodied in our taxonomy in following sections—could produce different recommendation results. Until such full analyses are conducted, the lack of a recommendation could be worse for society than a recommendation would be.

### Potential Impact of a Societal Perspective

The significance, and potentially decisive impact, of adopting a broad-benefits approach is illustrated by the UK's experience with MenB vaccination recommendation. The UK's official vaccine assessors, the Joint Committee on Vaccination and Immunisation (JCVI), first considered a 2013 CEA that found MenB vaccination likely to be cost-ineffective because of the disease's rarity (79). On that basis, JCVI failed to recommend the vaccine (80). However, health technology assessors recognized that their initial economic evaluation focused primarily on health-centric benefits and that acknowledging and measuring possible additional sources could potentially change this outcome.

The group solicited an updated analysis that considered a wider range of the vaccine's full benefits, including healthcare costs, litigation mitigation, and quality of life losses for caregivers in addition to those for patients themselves. Based on concerns that CEAs would not fully capture QALY losses, especially for children, or capture societal preferences for preventing especially severe diseases like MenB, the updated analysis also included a quality of life adjustment factor of 3×. The resulting analysis, performed by the same researchers as the previous analysis, found that at competitive prices the vaccine would likely be cost-effective for infants, an especially at-risk group (81). JCVI therefore reversed its position and recommended the vaccine for reimbursement for infants 2, 4, and 12 months of age, negotiating with manufacturers to procure the vaccine at a cost-effective price (80).

Ultimately, utilizing a broad benefits approach was enough to motivate a decisive change in vaccine policy from “no recommendation” to “recommendation” for infants. While this experience does not guarantee similar results in other settings, it indicates the risk a country runs in using a narrow framework. Had the UK failed to perform its broad benefits analysis, the country would not have reaped the benefits of MenB vaccination, which the later analysis suggests are substantial (82).

However, even though JCVI approached a broad framework by including additional sources of benefit, later public response to MenB vaccination policy in the UK suggests that this perspective is still not broad enough to capture all benefits fully. Its recommendation did not extend to children older than 12 months or to adolescents, for whom vaccination was still found to be cost-ineffective. In February 2016, the death of 2-year-old Faye Burdett and published pictures of her condition with MenB sparked an unprecedented public response, with more than 820,000 signatures added to a petition calling for vaccination recommendation and reimbursement up to age 11 (for reference, only 10,000 signatures are needed for a petition to receive a response from the government) (83). Around the same time, many individuals in the UK demanded the vaccine through the private market, financing it out-of-pocket. In fact, demand was so great that suppliers could not keep pace, resulting in reports of a shortage around January 2016 (84). This shortage was reported as alleviated around May 2016 (85). The JCVI convened in response, but failed to change its recommendation based on cost-effectiveness levels.

The 2016 response in the UK strongly suggests that policymakers' recommendations and societal preferences were misaligned. This implies that the subset of broad-perspective benefits that JCVI included in its CEA is not sufficient to accurately and fully capture the value of MenB vaccination. An incomplete benefits framework may well have resulted in JCVI failing to change their recommendation policy in response to public dissatisfaction.

Moving in the direction that JCVI did toward a societal perspective seems plausibly decisive in changing non-recommendations to recommendations in many settings (perhaps including the US), and could help resolve some of the challenges health technology assessors face in deciding how to treat interventions against rare, severe diseases. If expanding the scope of HTAs to consider a range of large societal benefits is indeed plausibly decisive, then failing to do so may result in an inefficient allocation of health budgets away from vaccines whose value isn't immediately obvious but nonetheless protect against diseases severe enough to impose substantial burdens. It may also avoid unnecessary preventable morbidity and mortality. However, as the UK's experience suggests, a fully comprehensive framework of benefits must consider more categories than traditionally estimated and must capture any societal preference for prioritizing especially severe diseases. There is need for a more long-term, systematic, and replicable solution to evaluations. In the following section, we propose a taxonomy that accounts for these considerations. It is worth reemphasizing here that while our discussion of each benefit focuses on MenB for the purposes of this paper, this adaptable framework can be readily applied to other meningococcal vaccines covering a broad set of disease serogroups (though the magnitude of benefits from each potential source may vary).

## A Taxonomy of the Full Benefits of MenB Vaccination

Our framework comprises a taxonomy of MenB vaccination's full benefits. In addition to distinguishing between traditionally captured narrow benefits and additional broad benefits, it sorts vaccination benefit categories along two other dimensions. The first dimension distinguishes between primarily health-related and primarily non-health-related benefits. The second dimension distinguishes between primarily internalized benefits (i.e., benefits that are enjoyed by the vaccine recipient or members of his or her household) and primarily externalized benefits (i.e., benefits that are enjoyed by anyone else). These two dichotomies are arranged on x- and y-axes, respectively. This creates a two-by-two matrix with four quadrants. For example, Quadrant I contains internalized health benefits, while Quadrant IV contains externalized non-health benefits.

Note that the assignment of benefits to quadrants is not rigid. For instance, certain benefits may be internalized in some situations and externalized in others, while others could be both at once. We categorize such ambiguous benefits according to our sense of what would be most salient for researchers and policymakers. But our categorizations should not be taken as definitive.

Admittedly, several of the benefits outlined in our taxonomy are likely empirically small given MenB's rarity. But, for the sake of completeness, we believe a full benefits framework should nevertheless count them. Since the same taxonomy can also be used to evaluate other severe diseases, there is additional value in creating a methodical, comprehensive framework that can be applied to numerous health technology assessments of infectious diseases. Table 1 presents our taxonomy.

**Table 1:** Taxonomy of the full benefits of MenB vaccination.

	**Health benefits**	**Non-health benefits**
**Internalized**	*Direct health gains* Household health externalities Prevention and amelioration of comorbidities Reductions in nosocomial infections **I**	Education gains Labor market productivity gains Non-market productivity and leisure gains Caregiver productivity and leisure gains Risk reduction gains **II**
**Externalized**	Full public health benefits **III**	*Healthcare cost savings* Social preference fulfillment Outbreak control gains Litigation mitigation Macroeconomic gains Institutional disruptions Equity gains Health system efficiency gains **IV**

*Italicized benefits categories comprise narrow benefits. All listed benefits are broad benefits*.

We now discuss each element within the taxonomy in turn.

### Quadrant I: Internalized Health Benefits

Quadrant I contains four categories of internalized health benefits: direct health gains, household health externalities, prevention and amelioration of comorbidities, and reductions in nosocomial infections.

#### Direct Health Gains

Direct health gains are perhaps the most obvious benefit of MenB vaccination. They represent the value the vaccine offers in preventing disease in the vaccine recipient, most often measured in QALYs or disability-adjusted life years (DALYs).

Because all CEAs of MenB vaccination, which often inform related policy decisions, necessarily measure the health impacts of vaccination (62–65, 86) we characterize this as one of our two narrow benefits. But while CEAs often capture this benefit, they have never captured it comprehensively. As discussed earlier, studies often analyze only health costs related to the acute disease phase rather than the health costs of long-term sequelae. A full benefits approach would include all disease outcomes and sequelae.

Moreover, vaccines targeted to a specific disease sometimes have effects on health outcomes that are not directly related to that disease. For example, some research finds that influenza vaccination among elderly adults may lower all-cause mortality (87, 88)^1^. So-called non-specific effects have also been demonstrated with a particular MenB vaccine: one recent study in New Zealand finds an association between administration of the MeNZB vaccine and reductions in gonorrhea diagnoses (89). A full accounting of vaccination benefits should count such non-specific effects.

In addition to lowering disease incidence, vaccines could also impact disease severity. This could happen by inducing better immune responses against the target disease, resulting in milder outcomes conditional on infection. Though no available literature documents such effects from MenB or any IMD vaccination and further investigation is needed, a full accounting of the vaccine's benefits would consider the possibility of this phenomenon.

Finally, insofar as MenB vaccination causes adverse events, these health costs should be counted against its health benefits.

#### Household Health Externalities

Our first non-narrow benefit category relates to health benefits of MenB vaccination that other members of the vaccine recipient's household enjoy. Perhaps the most readily apparent example of this category surrounds household-level herd protection. Because MenB is contagious, preventing MenB could plausibly prevent its spread to household contacts of the would-be patient (though current evidence on the potential magnitude of MenB vaccination herd effects is limited in the literature)^2^.

A vaccine recipient's household could enjoy further health benefits through additional mechanisms. Recall that Al-Janabi and colleagues demonstrate higher rates of ailments such as anxiety and depression and a commensurate QALY loss among household members living with a patient with severe IMD-related sequelae (60). This means that the vaccine could prevent not only MenB-related health burdens, but these other derivative household-level health burdens as well.

#### Prevention and Amelioration of Comorbidities

MenB patients can suffer additional health burdens in cases where MenB causes new comorbidities to develop or aggravates preexisting comorbidities. Thus, preventing MenB through vaccination would also prevent the development or worsening of these comorbidities and their associated health burdens. This represents another avenue through which MenB vaccines could confer health benefits on their recipients.

#### Reductions in Nosocomial Infections

Preventing MenB prevents MenB-related hospital visits, some of which could lead to the patient contracting a nosocomial infection (90). MenB vaccination could prevent not only the MenB infection, but also a secondary nosocomial infection, conferring further health benefits on the vaccine recipient.

Notably, case reports suggest that nosocomial IMD outbreaks are possible (91–93). Preventing an initial case through vaccination would stop the spread within a hospital, creating a health benefit for others in the hospital that should also be counted^3^.

### Quadrant II: Internalized Non-health Benefits

Quadrant II contains five internalized non-health benefit categories: education gains, labor market productivity gains, nonmarket productivity and leisure gains, caregiver productivity and leisure gains, and risk reduction gains.

#### Education Gains

Children who contract MenB can experience interruptions to education in at least three ways. First, they may miss days of school while sick (94). Second, they may experience worse cognitive functioning and therefore retain less while at school (12, 95). Third, largely because of these first two effects, they may experience lower overall educational attainment (96, 97). Because education is so instrumental in driving productivity and economic growth (98), these educational burdens can have costly adverse effects. The full value of preventing MenB therefore includes the long-term value of preventing these educational losses.

#### Labor Market Productivity Gains

While MenB's education-related costs can lead to losses in lifetime productivity, MenB can also impact productivity more directly when its burdens extend into working ages. MenB can lead to missed workdays and can hinder productivity while at work (94, 99). All of this can lead to missed opportunities for work-related skill development or career advancement. Of course, a death would wipe out the victim's entire lifetime's worth of market productivity. Preventing these productivity losses represents further potential benefits of MenB vaccination that deserve consideration by policymakers.

#### Non-market Productivity and Leisure Gains

In addition to market productivity, which involves paid work, MenB also impacts non-market productivity, which involves unpaid work. Specifically, MenB can impact patients' ability to spend time volunteering, caregiving, and doing housework and can impact their productivity while working on such activities (99).

Moreover, MenB affects not only productive time, but also leisure time, or all non-productive time besides self-care. This includes activities like socializing or watching television, but not, for example, sleeping or grooming. These represent other benefits that analyses of MenB vaccination should consider.

#### Caregiver Productivity and Leisure Gains

When vaccination prevents MenB, the patient is not the only one who enjoys productivity- and leisure-related benefits. Any would-be caregivers—often family members^4^—also avoid the potentially catastrophic effects that MenB can have on the quantity and quality of their productive time and leisure time (100, 101). The value of avoiding these costs to caregivers should also be counted.

#### Risk Reduction Gains

The last internalized non-health benefit concerns reductions in risk-related costs of MenB. These costs fall into two main categories. First, as discussed previously, MenB can impose severe health and financial burdens on individuals who contract the disease and their close contacts (60, 100). Risk-averse individuals are demonstrably willing to pay to reduce such risks generally (102). In this sense, MenB vaccination reduces health risk and has financial benefits similar to an insurance policy in that it helps to smooth health spending over time.

Second, community members may feel anxiety or dread during a MenB outbreak, especially given the high unpredictability of disease incidence (103). If vaccination prevents or mitigates the harms of such an outbreak, then these avoided negative emotions should count as a benefit.^5^ In addition to avoided negative emotions, some individuals may feel a sense of security and peace of mind immediately after vaccination from knowing that they are protected against the disease (referred to as “utility in anticipation”) (104, 105). This sense of security raises quality of life and should ideally be incorporated in measures of risk reduction gains.

### Quadrant III: Externalized Health Benefits

Quadrant III contains only one externalized health benefit category, but it is a comprehensive one: full public health benefits.

Recent lines of inquiry have sought to expand the range of public health benefits that authorities consider when making vaccination-related decisions. This broad range of health benefits is known as the full public health value (FPHV) of vaccination (3). This encompasses all health-related benefits broader society enjoys when an individual is vaccinated.

The first such public health benefit concerns the herd protection that non-vaccinated community members experience. This is the phenomenon by which vaccination prevents disease carriage and acquisition, thus preventing the spread of the disease. While some evidence of herd protection following vaccination against IMD serogroups C and A exists (106–108), such evidence has not yet been demonstrated for serogroup B vaccination (109). But should such evidence be discovered for MenB vaccination, analyses should consider it (110). Herd effects would likely be highly impactful for those groups at higher risk, such as college students and military personnel (111). For the same reasons underlying their increased risk factors (living in close proximity with frequent and prolonged contact), the benefits of herd immunity would be especially pronounced within these high-incidence groups and help slow transmission to the general population. Failing to account for herd effects can lead to underestimation of external benefits.

A second aspect of the FPHV of meningococcal vaccination in general has to do with antimicrobial resistance (AMR). As antimicrobials are used to treat and prevent disease, bacteria can develop resistance (112). The prevalence of AMR related to IMD serogroups appears limited (113) (and we are not aware of any evidence specific to serogroup B AMR), but the development of antibiotic resistance in Neisseria meningitidis has been reported in certain settings (114). Even if meningococcus does not develop resistance, exposure to antibiotics also risks the development of AMR in non-target pathogens^6^. Using vaccines, and thereby precluding the need to use antimicrobials for treatment or for chemoprophylaxis (115–117), can help slow the pace of AMR development among IMD pathogens and any other pathogen (including those that are carried asymptomatically) that would have been exposed to the antimicrobial (118). While no evidence is available of this effect following vaccination against IMD, substantial evidence exists involving a vaccine against pneumococcal disease, another bacterial disease (119–122).

### Quadrant IV: Externalized Non-health Benefits

The final quadrant comprises eight externalized non-health benefit categories: healthcare cost savings, social preference fulfillment, outbreak control gains, litigation mitigation, macroeconomic gains, institutional disruptions, equity gains, and health system efficiency gains.

#### Healthcare Cost Savings

Our second and last narrow benefit concerns the healthcare cost savings afforded by vaccination. Preventing IMD prevents its direct treatment costs, which can be immense in both developed and developing countries (123–126). Public payers often bear these costs, in which case these benefits are externalized, but they can also be internalized whenever medical expenses are paid out-of-pocket.

While this benefit category is categorized as narrow because policymakers often consider it when making MenB-related policy decisions, analyses rarely count the benefit comprehensively. The benefit should include direct costs incurred both during the acute disease phase and during any long-term medical treatments. As discussed previously, many analyses include only acute phase-related costs (45). Furthermore, this category should comprehensively count all relevant direct costs, including those for ambulance travel, drugs, medical devices, therapy, and everything else related to disease recovery and rehabilitation. But no available analyses count all such costs.

#### Social Preference Fulfillment

Vaccination against MenB derives value from the alignment between its effects and societal preferences. As discussed previously, a budding literature suggests that individuals are willing to pay disproportionate amounts to prevent severe disease (127, 128). This suggests a societal preference for prioritizing severe diseases over many milder diseases. Fulfilling that preference represents another benefit of MenB vaccination.

Similarly plausible is that MenB vaccination fulfills societal preferences related to prioritizing benefits that accrue to certain age groups (e.g., the very young) or to certain social groups (e.g., the military) that are at disproportionate risk for MenB.

#### Outbreak Control Gains

MenB outbreaks impose costs beyond those of the resulting diseases. These include spending on chemoprophylaxis; vaccine-related shipment, storage, administration, and labor; strains placed on first responders and emergency medical providers and the associated downstream effects; public education and messaging; and increased use of precautionary medical tests. As discussed earlier, Anonychuk and colleagues show that these costs can be immense (61). If vaccines prevent or mitigate the effects of outbreaks, analyses should consider the resulting outbreak-related cost savings.

#### Litigation Mitigation

Because misdiagnosing MenB is so common (52), medical lawsuits surrounding the disease are also common in some settings (129). When MenB is prevented, such lawsuits and their associated costs are also prevented. The value of preventing this litigation should include reduced payouts from medical lawsuits (81); lower malpractice insurance premiums for physicians; and the opportunity costs of time spent by judges, lawyers, clients, doctors, court staff, and others related to the case.

Note that the value of these benefits may overlap with other costs. For example, a lawsuit payout from a doctor may cover medical costs for a patient's family, which overlaps with the vaccine's value in reducing healthcare costs. When calculating the value of preventing litigation-related costs, care should be taken not to double-count benefits that overlap with other benefit categories^7^.

#### Macroeconomic Gains

The next benefit category included in our taxonomy has to do with macroeconomic benefits. As poor health hinders economic growth (130), improving health through vaccination improves such growth. Valuing these gains should capture benefits to macroeconomic growth through the productivity gains previously outlined. It should also include the value of poverty reduction insofar as disease creates and sustains poverty. Furthermore, disease can hinder tourism, including routine tourism and tourism during special events like the Hajj or the Olympics (4). Finally, poor health is associated with lower foreign direct investment inflows (131).

Given the rarity of MenB, such high-level macroeconomic gains from MenB vaccination are likely marginal. Potential sub-macro community gains, however, may be larger and worth additional consideration, such as the avoided costs of an outbreak for a university or military base.

#### Institutional Disruptions

While MenB outbreaks are rare (only 2–3 out of every 100 cases are related to outbreaks in the US) (132), organizations facing an outbreak may be forced to contend with substantial and costly disruptions. There have been at least seven outbreaks on US college campuses between 2013 and 2019 (132, 133)^8^. These well-publicized outbreaks cause considerable disruption and give rise to an impression of increased risk among students, faculty, and parents (134). In response to outbreaks, affected universities have coordinated mass vaccination efforts with local health authorities, organizing emergency clinics and follow-up clinics to administer MenB vaccines to students and faculty. Administrators of affected grade schools may need to cancel classes and extracurricular activities in response to meningococcal outbreaks, and parents may pull their children from schools that remain open for fear of infection (135, 136). This type of disruption costs the institutions resources (and potentially negative press attention), costs students time in the classroom, and may cost parents time, money, and energy to care for children who are not at school. Organization-based MenB cases and outbreaks have also been reported in childcare centers, military groups, and correctional facilities, which may similarly experience costly disruptions (137, 138).

While the aggregate institutional disruption is likely quite small given MenB's rarity, high incidence rates within an organization could nevertheless raise dissatisfaction levels and erode trust in the governing institution. The debilitating outcomes of severe diseases like MenB may raise concerns about the capability and accountability of leaders, who may be perceived as misallocating resources and failing to provide necessary services that should protect the citizenry. This erosion of trust is one additional potential consequence of institution-based outbreaks.

The overall costs of a MenB outbreak to institutional stability may be slight compared to other costs of the disease, but should nonetheless be accounted for in a complete consideration of all vaccine benefits.

#### Equity Gains

High medical expenditures associated with treatment of vaccine-preventable diseases like MenB can generate substantial financial stress for affected households. This burden may be especially heavy for low-income or uninsured families who pay for many of these costs out-of-pocket, particularly in developing countries. These costs, in addition to potential costs of lost time and wages, can drive households into a cycle of poverty from which it is difficult to emerge. Such household medical impoverishment due to treatment costs can widen health and financial equity gaps within and between communities.

This concept is broadly applicable to IMD and other infectious diseases in general, which tends to fall disproportionately on lower-income populations (139, 140). This is in part due to other factors associated with lower socioeconomic status that can exacerbate the severity and cost of the disease for poorer individuals, such as undernutrition, lack of access to clean water, lack of access to necessary care, and lower hygiene and sanitation (141). Research has shown that vaccination on average therefore confers relatively greater benefits on the poor than on middle- and high-income groups. Since vaccine benefits accrue predominantly to the lowest income groups in certain settings, policymakers should be informed of the considerable distributional impact vaccines can have for health and economics and understand their potential benefits as tools for promoting health equity (142).

#### Health System Efficiency Gains

The last benefit category connects MenB vaccination and the efficiency of healthcare systems. Fewer MenB cases means medical supplies and professionals' time can be reallocated to other patients, resulting in a more efficient allocation of hospital resources and increased capacity to treat other conditions. Given the severity of MenB and the substantial time and care demands involved in its treatment, the financial and medical resources saved by each prevented case are likely sizeable. Vaccination also leads to reduced consumption of medication (143), such as the antibiotics involved in MenB treatment. This not only helps to ease the strain on hospital budgets, but also benefits other groups of patients as hospitals can refocus their attention on unmet medical needs (143). Time and energy not spent diagnosing and treating MenB patients could even be spent instead on research and development efforts. Overall, preventing MenB cases can help marginally increase the sustainability of the healthcare system.

## Measurement of These Benefits

A formidable obstacle to implementing the above taxonomy is identifying measurement methods that can accurately capture all of these effects. While there are well-established approaches to measuring certain benefits (such as avoided hospitalization and medication costs, for example), others such as risk reduction gains and societal preferences are more challenging to estimate. JCVI's inclusion of a QALY adjustment factor (3×) in their economic evaluation of MenB, while a constructive effort to acknowledge and account for the shortcomings of current CEA tools, is too arbitrary an approach to capture vaccine benefits adequately and accurately. A more theoretically and empirically-grounded solution to increasing the precision of CEAs is required moving forward. We now discuss a set of possible measurement solutions and their relative advantages and disadvantages.

Perhaps the best way to measure MenB vaccination's benefits is to work such measurements into existing randomized control trials (RCTs). This may be performed, for example, by administering an economic questionnaire to RCT participants. However, these trials are often costly and logistically complex even without incorporating an economic component.

Absent the ability to work through existing RCTs, observational or registry-based studies represent another means of measuring these broader benefits of MenB vaccination (110). When done correctly, these studies offer the advantage of generating a reliable empirical basis for estimating the magnitude of these benefits.

But observational or registry-based studies face the challenges previously discussed in the context of existing CEAs' shortcomings: that MenB is often too rare to derive reliable parameters without a perhaps prohibitively large sample size. This rarity presents another reason in favor of post-implementation surveillance. Such surveillance would naturally identify all or nearly all MenB patients in a population. These MenB patients could then serve as a comparison group in a post-implementation observational study that measures one or more of these broad benefits. Because they would be identified from the entire relevant population, a large sample (relative to those from current observational studies) of MenB cases would be available for analysis. Surveillance programs have been implemented in many countries, but these have not generally captured vaccination's broad benefits (106, 144, 145). Surveillance programs can be improved and expanded to capture broad benefit categories, and this surveillance should continue over the long term to capture benefits that manifest over several years.

Before such studies are performed, another viable approach to estimating the magnitude of these benefits to a first approximation involves invoking estimates of the burdens of similar disease outcomes from other diseases. For example, absent reliable data on the full costs of MenB-derived hearing loss, one could examine analyses of the social and economic implications of hearing loss from other causes. While reliable MenB-specific, or at least IMD-specific, estimates are preferable, economic analyses can use these kinds of “proxy” estimates before studies produce high-quality IMD-related data. Appendix 1 lists references to many such “proxy” studies.

## Conclusion

HTAs as currently conducted often address the rarity of MenB, but fail to address its severity adequately. Accounting for the full range of costs that MenB vaccination can prevent will be important to addressing this issue. The taxonomy we present aligns with a general trend in the literature away from a narrow focus and can help guide the implementation of such a remedy. Prominent researchers, such as Martinón-Torres, are similarly identifying gaps in underreported burdens of IMD. He notes that “lifelong cognitive deficits, psychological stress, adaptive measures for reintegration into society, familial impact, and legal costs are systematically overlooked” (45). Our analysis lays out these systematically overlooked aspects and identifies benefits that policymakers would do well to consider in future evaluations of vaccines for MenB, IMD, and other infectious diseases.

This new framework provides some apparent directions for future research. First and perhaps most obvious is to quantify empirically in monetary terms as many of these benefits as is feasible. Where small sample sizes preclude empirical measurement, modeling or data from “proxy” diseases (see Appendix 1) can be used. Once quantified, these benefits should be included in analyses of the value of vaccination. Further research could explore the optimal methodology for assessing the full value-for-money proposition that vaccination offers. CEAs may not present the ideal means through which to capture broad benefits. Cost-benefit analyses may offer a superior framework^9^. Conducting both such analyses and comparing the different policy conclusions reached under each could illuminate the extent to which different methodologies impact policy recommendations.

Future research could also involve the application of the broad benefit principles to wider vaccination policy decisions for certain groups. For example, colleges and universities often require students to receive certain vaccines as a condition of attendance, and countries sometimes require specific vaccines for certain travelers. For instance, Saudi Arabia requires that Hajj and Umrah pilgrims receive several vaccines (146). Examining the potential role of meningococcal vaccines in such systems could help avoid costly outbreaks in these settings.

Ultimately, as vaccines become increasingly expensive, adopting a societal perspective to account for vaccination's full range of benefits and devising innovative ways to measure their impacts, will be critical to sound recommendation and reimbursement decisions. The conclusions we draw here are not only relevant to MenB, but will have implications for interventions against other rare, severe diseases as well. Failing to account fully for these costs could result in underinvestment in MenB vaccines' development, production, and delivery, with unnecessary health and economic burdens as an unfortunate result.

## Author Contributions

All authors made substantial contributions to the conception and design of the paper, the acquisition of the data, and drafting and critically revising the article.

## Conflict of Interest

AS was a full-time employee of DfD during the development and drafting of the manuscript. PK was a full-time employee of DfD. DB was a paid consultant to DfD and a member of the faculty at the Harvard T.H. Chan School of Public Health. He has received grant support from the Bill and Melinda Gates Foundation, the National Institute of Aging, and the World Health Organization. He has also received grant support and/or personal fees from Merck, Pfizer, GSK, Sanofi Pasteur, Sanofi Pasteur-MSD, and Gilead Life Sciences. LH was a full-time employee and a shareholder of Pfizer Inc.

## Footnotes

^1^For a summary of the literature on this topic, including arguments that such benefits are overstated, see Simonsen et al. (88).

^2^We are aware of no evidence surrounding intra-household herd effects from vaccination against any IMD serogroup, but we discuss evidence surrounding community-wide herd effects later in Quadrant III.

^3^While this benefit category is in Quadrant I, implying that it is primarily perceived as an internalized health benefit, this specific manifestation of the benefit would be externalized. This illustrates that benefit categories can be fluid with respect to their quadrant assignments.

^4^Formal caregivers from outside the household may also face an opportunity cost of time spent with MenB patients to the extent that it prevents their performing other work. That would be an externalized benefit, again illustrating that our assignments of benefit categories to quadrants are not rigid.

^5^Note that this last category is primarily an externalized benefit, which may belong in Quadrant IV. This is again illustrative of our observation that these quadrants are not rigid.

^6^WHO guidelines, for instance, caution that the prophylactic use of Rifampicin for household contacts of IMD patients may not be advisable in the African meningitis belt where Rifampicin is particularly important for tuberculosis treatment. World Health Organization (37).

^7^The same is true of all other benefit categories that may overlap. For example, consider an economic evaluation that assesses willingness to pay to avoid the QALY losses of MenB. Because QALYs assess disease severity and because people's perceptions of severity may depend on the extent to which the disease imposes constraints on, say, market work, the willingness to pay to avoid QALY losses may already capture the value of lost market work. Adding the value of lost market work to the willingness-to-pay figure would therefore double-count certain benefits.

^8^This includes outbreaks at the University of California, Santa Barbara (2013), Princeton University (2013–2014), Providence College (2015), University of Oregon (2015), Santa Clara University (2016), San Diego State University (2018), the Five College Consortium (2017–2018), and Columbia University (2019).

^9^For a brief discussion of the advantages of a benefit-cost analysis over a CEA, see the section entitled “Comparison of Benefits and Costs” in Bärnighausen et al. (6).

## Abbreviations

ACIP: Advisory Committee on Immunization Practices
AMR: Antimicrobial resistance
CEA: Cost-effectiveness analysis
DALY: Disability-adjusted life year
FPHV: Full public health value
HAS: Haute Autorité de Santé
HTA: Heath technology assessment
IMD: Invasive meningococcal disease
JCVI: Joint Committee on Vaccination and Immunisation
MD: Meningococcal disease
MenB: Serogroup B meningococcal disease
QALY: Quality-adjusted life year.
