# Supplementary material for: Health Technology Assessment for Vaccines Against Rare, Severe Infections: Properly Accounting for Serogroup B Meningococcal Vaccination's Full Social and Economic Benefits
Source: Front Public Health. 2020 Jul 10;8:261. doi: 10.3389/fpubh.2020.00261 (PMC7366491; doi:10.3389/fpubh.2020.00261)
Supplement: Supplementary file 1 [file Table_1.DOCX]

Supplementary Material

# Appendix 1: Studies that Report the Social and Economic Costs of “Proxy” Diseases

This appendix lists studies that report the social and economic costs of “proxy” diseases that approximate many of IMD’s long-term sequelae. We organize the references by sequela.

- Hearing loss
  - Mohr PE, Feldman JJ, Dunbar JL, McConkey-Robbins A, Niparko JK, Rittenhouse RK, Skinner MW. The societal costs of severe to profound hearing loss in the United States. International Journal of Technology Assessment in Health Care. 2000;16(4):1120–35.
  - Bond M, Mealing S, Anderson R, Elston J, Weiner G, Taylor RS, et al. The effectiveness and cost-effectiveness of cochlear implants for severe to profound deafness in children and adults: a systematic review and economic model. Health Technology Assessment. 2009;13(44):i–330.
  - Wallhagen MI, Strawbridge WJ, Shema SJ, Kaplan GA. Impact of self-assessed hearing loss on a spouse: a longitudinal analysis of couples. The Journals of Gerontology Series B: Psychological Sciences and Social Sciences. 2004;59(3):S190–6.
  - Tambs K. Moderate effects of hearing loss on mental health and subjective well-being: results from the Nord-Trøndelag Hearing Loss Study. Psychosomatic Medicine. 2004;66(5):776–82.
  - Ciorba A, Bianchini C, Pelucchi S, Pastore A. The impact of hearing loss on the quality of life of elderly adults. Clinical Interventions in Aging. 2012;7:159–63.
  - Hintermair M. Self-esteem and satisfaction with life of deaf and hard-of-hearing people—a resource-oriented approach to identity work. Journal of Deaf Studies and Deaf Education. 2007;13(2):278–300.
  - Centers for Disease Control and Prevention. Economic costs associated with mental retardation, cerebral palsy, hearing loss, and vision impairment—United States, 2003. Morbidity and Mortality Weekly Report. 2004;53(3):57–9.
- Amputations
  - Margolis DJ, Malay DS, Hoffstad OJ, Leonard CE, MaCurdy T, Tan Y, et al. Economic burden of diabetic foot ulcers and amputations: data points #3. In: Data Points Publication Series [Internet]. Rockville (MD): Agency for Healthcare Research and Quality (US); 2011. https://www.ncbi.nlm.nih.gov/books/NBK65152/.
  - Driver VR, Fabbi M, Lavery LA, Gibbons G. The costs of diabetic foot: the economic case for the limb salvage team. Journal of Vascular Surgery. 2010;52(3):17S–22S.
  - Ragnarson Tennvall G, Apelqvist J. Health-economic consequences of diabetic foot lesions. Clinical Infectious Diseases. 2004;39(Supplement 2):S132–9.
  - Ortegon MM, Redekop WK, Niessen LW. Cost-effectiveness of prevention and treatment of the diabetic foot. Diabetes Care. 2004;27(4):901–7.
- Neurological deficits
  - Hewer RL. The economic impact of neurological illness on the health and wealth of the nation and of individuals. Journal of Neurology, Neurosurgery & Psychiatry. 1997;63(Supplement 1):S19–23.
  - Mar J, Arrospide A, Begiristain JM, Larrañaga I, Elosegui E, Oliva-Moreno J. The impact of acquired brain damage in terms of epidemiology, economics and loss in quality of life. BMC Neurology. 2011;11(1):46.
  - Bailey Jr. DB, Raspa M, Bishop E, Mitra D, Martin S, Wheeler A, Sacco P. Health and economic consequences of fragile X syndrome for caregivers. Journal of Developmental & Behavioral Pediatrics. 2012;33(9):705–12.
- Musculoskeletal disorders
  - Morse TF, Dillon C, Warren N, Levenstein C, Warren A. The economic and social consequences of work-related musculoskeletal disorders: the Connecticut Upper-Extremity Surveillance Project (CUSP). International Journal of Occupational and Environmental Health. 1998;4(4):209–16.
  - Lee P. The economic impact of musculoskeletal disorders. Quality of Life Research. 1994;3(Supplement 1):S85–91.
  - Coyte PC, Asche CV, Croxford R, Chan B. The economic cost of musculoskeletal disorders in Canada. Arthritis & Rheumatology. 1998;11(5):315–25.
  - Lindgren B. The economic impact of musculoskeletal disorders. Acta Orthopaedica Scandinavica. 1998;69(Supplement 281):58–60.
  - Schultz IZ, Stowell AW, Feuerstein M, Gatchel RJ. Models of return to work for musculoskeletal disorders. Journal of Occupational Rehabilitation. 2007;17(2):327–52.
  - Lanes SF, Lanza LL, Radensky PW, Yood RA, Meenan RF, Walker AM, Dreyer NA. Resource utilization and cost of care for rheumatoid arthritis and osteoarthritis in a managed care setting: the importance of drug and surgery costs. Arthritis & Rheumatology. 1997;40(8):1475–81.
  - Dall TM, Gallo P, Koenig L, Gu Q, Ruiz D. Modeling the indirect economic implications of musculoskeletal disorders and treatment. Cost Effectiveness and Resource Allocation. 2013;11(1):5.
  - Hunter DJ, Schofield D, Callander E. The individual and socioeconomic impact of osteoarthritis. Nature Reviews Rheumatology. 2014;10(7):437–41.
  - Abbott JH, Usiskin IM, Wilson R, Hansen P, Losina E. The quality-of-life burden of knee osteoarthritis in New Zealand adults: a model-based evaluation. PLOS ONE. 2017;12(10):e0185676.
  - Dickens C, McGowan L, Clark-Carter D, Creed F. Depression in rheumatoid arthritis: a systematic review of the literature with meta-analysis. Psychosomatic Medicine. 2002;64(1):52–60.
- Organ failure
  - Black C, Sharma P, Scotland G, McCullough K, McGurn D, Robertson L, et al. Early referral strategies for management of people with markers of renal disease: a systematic review of the evidence of clinical effectiveness, cost-effectiveness and economic analysis. Health Technology Assessment. 2010;14(21):1–84.
  - Ambrosy AP, Fonarow GC, Butler J, Chioncel O, Greene SJ, Vaduganathan M, et al. The global health and economic burden of hospitalizations for heart failure: lessons learned from hospitalized heart failure registries. Journal of the American College of Cardiology. 2014;63(12):1123–33.
  - Modell V, Gee B, Lewis DB, Orange JS, Roifman CM, Routes JM, Sorensen RU, Notarangelo LD, Modell F. Global study of primary immunodeficiency diseases (PI)—diagnosis, treatment, and economic impact: an updated report from the Jeffrey Modell Foundation. Immunologic research. 2011 Oct 1;51(1):61–70.
  - Bleicken B, Hahner S, Loeffler M, Ventz M, Decker O, Allolio B, Quinkler M. Influence of hydrocortisone dosage scheme on health‐related quality of life in patients with adrenal insufficiency. Clinical Endocrinology. 2010;72(3):297–304.
  - Ekman B, Bachrach‐Lindström M, Lindström T, Wahlberg J, Blomgren J, Arnqvist HJ. A randomized, double‐blind, crossover study comparing two‐and four‐dose hydrocortisone regimen with regard to quality of life, cortisol and ACTH profiles in patients with primary adrenal insufficiency. Clinical Endocrinology. 2012;77(1):18–25.
  - Tiemensma J, Andela CD, Kaptein AA, Romijn JA, van der Mast RC, Biermasz NR, Pereira AM. Psychological morbidity and impaired quality of life in patients with stable treatment for primary adrenal insufficiency: cross-sectional study and review of the literature. European Journal of Endocrinology. 2014;171(2):171–82.
- Seizures
  - Birbeck G, Chomba E, Atadzhanov M, Mbewe E, Haworth A. The social and economic impact of epilepsy in Zambia: a cross-sectional study. The Lancet Neurology. 2007;6(1):39–44.
  - Yoon D, Frick KD, Carr DA, Austin JK. Economic impact of epilepsy in the United States. Epilepsia. 2009;50(10):2186–91.
  - Allers K, Essue BM, Hackett ML, Muhunthan J, Anderson CS, Pickles K, et al. The economic impact of epilepsy: a systematic review. BMC Neurology. 2015;15(1):245.
  - Hermann B, Jacoby A. The psychosocial impact of epilepsy in adults. Epilepsy & Behavior. 2009;15(2):S11–6.
  - Valeta T. Psychosocial impact of epilepsy. In: The epilepsy book: a companion for patients. 2017 (p. 161–6). Springer, Cham.
  - Rodenburg R, Wagner JL, Austin JK, Kerr M, Dunn DW. Psychosocial issues for children with epilepsy. Epilepsy & Behavior. 2011;22(1):47–54.
  - Quintas R, Raggi A, Giovannetti AM, Pagani M, Sabariego C, Cieza A, Leonardi M. Psychosocial difficulties in people with epilepsy: a systematic review of literature from 2005 until 2010. Epilepsy & Behavior. 2012;25(1):60–7.
  - Gandy M, et al. The psychosocial correlates of depressive disorders and suicide risk in people with epilepsy. Journal of Psychosomatic Research 2013;74(3):227–32.
- Ataxia
  - López-Bastida J, Perestelo-Pérez L, Montón-Alvarez F, Serrano-Aguilar P. Social economic costs and health-related quality of life in patients with degenerative cerebellar ataxia in Spain. Movement Disorders. 2008;23(2):212–7.
  - Pike J, Jones E, Rajagopalan K, Piercy J, Anderson P. Social and economic burden of walking and mobility problems in multiple sclerosis. BMC Neurology. 2012;12(1):94.
  - Wilson CL, Fahey MC, Corben LA, Collins VR, Churchyard AJ, Lamont PJ, Delatycki MB. Quality of life in Friedreich ataxia: what clinical, social and demographic factors are important? European Journal of Neurology. 2007;14(9):1040–7.
- Hemiplegia
  - Tonmukayakul U, Shih S, Bourke-Taylor H, Imms C, Reddihough D, Cox L et al. Systematic review of the economic impact of cerebral palsy. Research in Developmental Disabilities. 2018;80:93–101.
  - Ottomanelli L, Lind L. Review of critical factors related to employment after spinal cord injury: implications for research and vocational services. The Journal of Spinal Cord Medicine. 2009;32(5):503–31.
  - Michelsen SI, Uldall P, Kejs AM, Madsen M. Education and employment prospects in cerebral palsy. Developmental Medicine and Child Neurology. 2005;47(8):511–7.
  - Saeki S, Toyonaga T. Determinants of early return to work after first stroke in Japan. Journal of Rehabilitation Medicine. 2010;42(3):254–8.
  - Varni JW, Limbers CA, Burwinkle TM. Impaired health-related quality of life in children and adolescents with chronic conditions: a comparative analysis of 10 disease clusters and 33 disease categories/severities utilizing the PedsQL™ 4.0 Generic Core Scales. Health and Quality of Life Outcomes. 2007;5(1):43.
